# Supplementary material for: In Silico Structure-Based Approach for Group Efficiency Estimation in Fragment-Based Drug Design Using Evaluation of Fragment Contributions
Source: Molecules. 2022 Mar 18;27(6):1985. doi: 10.3390/molecules27061985 (PMC8951103; doi:10.3390/molecules27061985)
Supplement: Supplementary file 1 [file molecules-27-01985-s001.zip › molecules-1600187-supplementary.pdf]

Supplementary Materials

# In Silico Structure-Based Approach for Group Efficiency Estimation in Fragment-Based Drug Design Using Evaluation of Fragment Contributions

**Dmitry A. Shulga, Nikita N. Ivanov and Vladimir A. Palyulin**

Table S1. Values of scoring function AutoDock 4 obtained by scoring of compound **1** fragments with experimental geometry in the binding sites of target models (PDB:3IUE)

| Fragment | E, kcal/mol | Share, % | NH | GE[1] | GE[2] |
|---|---|---|---|---|---|
| **1** | -1.68 | 15.5% | 7 | 0.24 | **0.18** |
| **2** | -1.62 | 15.0% | 6 | 0.27 | **0.20** |
| **3** | -4.82 | 44.5% | 11 | 0.44 | **0.32** |
| **4** | -2.71 | 25.0% | 4 | 0.68 | **0.50** |
| *sum:* | *-10.83* | *100.0%* | - | - | - |

**NH** - number of non hydrogen (heavy) atoms.
**GE[1]** - group efficiency calculated as a ratio of scoring function value and number of non hydrogen atoms.
**GE[2]** - group efficiency (see *Eq. 4*) calculated as a ratio of the product of fragment share and experimental binding energy (see *Eq. 2*) and number of non hydrogen atoms.

Table S2. Values of scoring function AutoDock 4 (Vina 1.2) obtained by scoring of compound **1** fragments with experimental geometry in the binding sites of target models (PDB:3IUE)

| Fragment | E, kcal/mol | Share, % | NH | GE[1] | GE[2] |
|---|---|---|---|---|---|
| **1** | -1.68 | 15.9% | 7 | 0.24 | **0.18** |
| **2** | -1.43 | 13.6% | 6 | 0.24 | **0.18** |
| **3** | -4.75 | 44.9% | 11 | 0.43 | **0.32** |
| **4** | -2.71 | 25.6% | 4 | 0.68 | **0.51** |
| *sum:* | *-10.58* | *100.0%* | - | - | - |

**NH** - number of non hydrogen (heavy) atoms.
**GE[1]** - group efficiency calculated as a ratio of scoring function value and number of non hydrogen atoms.
**GE[2]** - group efficiency (see *Eq. 4*) calculated as a ratio of the product of fragment share and experimental binding energy (see *Eq. 2*) and number of non hydrogen atoms.

Table S3. Values of scoring function AutoDock Vina 1.1.2 obtained by scoring of compound **1** fragments with experimental geometry in the binding sites of target models (PDB:3IUE)

| Fragment | E, kcal/mol | Share, % | NH | GE[1] | GE[2] |
|---|---|---|---|---|---|

| | | | | | |
|---|---|---|---|---|---|
| **1** | -2.15 | 17.8% | 7 | 0.31 | **0.20** |
| **2** | -2.24 | 18.5% | 6 | 0.37 | **0.25** |
| **3** | -5.45 | 45.0% | 11 | 0.50 | **0.33** |
| **4** | -2.26 | 18.7% | 4 | 0.57 | **0.37** |
| *sum:* | *-12.11* | *100.0%* | - | - | - |

**NH** - number of non hydrogen (heavy) atoms.
**GE**$^{1}$ - group efficiency calculated as a ratio of scoring function value and number of non hydrogen atoms.
**GE**$^{2}$ - group efficiency (see *Eq. 4*) calculated as a ratio of the product of fragment share and experimental binding energy (see *Eq. 2*) and number of non hydrogen atoms.

Table S4. Values of scoring function AutoDock Vina 1.2.3 obtained by scoring of compound **1** fragments with experimental geometry in the binding sites of target models (PDB:3IUE)

| **Fragment** | **E, kcal/mol** | **Share, %** | **NH** | **GE**$^{1}$ | **GE**$^{2}$ |
|---|---|---|---|---|---|
| **1** | -2.15 | 17.8% | 7 | 0.31 | **0.20** |
| **2** | -2.24 | 18.5% | 6 | 0.37 | **0.25** |
| **3** | -5.45 | 45.0% | 11 | 0.50 | **0.33** |
| **4** | -2.26 | 18.7% | 4 | 0.57 | **0.37** |
| *sum:* | *-12.11* | *100.0%* | - | - | - |

**NH** - number of non hydrogen (heavy) atoms.
**GE**$^{1}$ - group efficiency calculated as a ratio of scoring function value and number of non hydrogen atoms.
**GE**$^{2}$ - group efficiency (see *Eq. 4*) calculated as a ratio of the product of fragment share and experimental binding energy (see *Eq. 2*) and number of non hydrogen atoms.

Table S5. Values of scoring function Vinardo (Vina 1.2.3) obtained by scoring of compound **1** fragments with experimental geometry in the binding sites of target models (PDB:3IUE)

| **Fragment** | **E, kcal/mol** | **Share, %** | **NH** | **GE**$^{1}$ | **GE**$^{2}$ |
|---|---|---|---|---|---|
| **1** | -1.17 | 14.0% | 7 | 0.17 | **0.16** |
| **2** | -1.22 | 14.6% | 6 | 0.20 | **0.19** |
| **3** | -3.57 | 42.8% | 11 | 0.32 | **0.31** |
| **4** | -2.37 | 28.5% | 4 | 0.59 | **0.57** |
| *sum:* | *-8.34* | *100.0%* | - | - | - |

**NH** - number of non hydrogen (heavy) atoms.
**GE**$^{1}$ - group efficiency calculated as a ratio of scoring function value and number of non hydrogen atoms.
**GE**$^{2}$ - group efficiency (see *Eq. 4*) calculated as a ratio of the product of fragment share and experimental binding energy (see *Eq. 2*) and number of non hydrogen atoms.

Table S6. Values of scoring function ΔVinaRF20 obtained by scoring of compound **1** fragments with experimental geometry in the binding sites of target models (PDB:3IUE)

| **Fragment** | **$pK_d$** | **E, kcal/mol*** | **Share, %** | **NH** | **GE[1]** | **GE[2]** |
|---|---|---|---|---|---|---|
| **1** | 1.61 | -2.21 | 18.1% | 7 | 0.32 | **0.20** |
| **2** | 1.79 | -2.45 | 20.1% | 6 | 0.41 | **0.27** |
| **3** | 4.20 | -5.75 | 47.1% | 11 | 0.52 | **0.34** |
| **4** | 1.31 | -1.79 | 14.7% | 4 | 0.45 | **0.29** |
| *sum:* | - | *-12.21* | *100.0%* | - | - | - |

**NH** - number of non hydrogen (heavy) atoms.
**GE[1]** - group efficiency calculated as a ratio of scoring function value and number of non hydrogen atoms.
**GE[2]** - group efficiency (see *Eq. 4*) calculated as a ratio of the product of fragment share and experimental binding energy (see *Eq. 2*) and number of non hydrogen atoms.
*** E, kcal/mol** = $pK_d$/-0.73

Table S7. Values of scoring function DrugScore X obtained by scoring of compound **1** fragments with experimental geometry in the binding sites of target models (PDB:3IUE)

| **Fragment** | **log p** | **E, kcal/mol*** | **Share, %** | **NH** | **GE[1]** | **GE[2]** |
|---|---|---|---|---|---|---|
| **1** | -16.29 | -9.77 | 13.4% | 7 | 1.40 | **0.15** |
| **2** | -12.06 | -7.24 | 9.9% | 6 | 1.21 | **0.13** |
| **3** | -75.08 | -45.05 | 61.7% | 11 | 4.10 | **0.45** |
| **4** | -18.19 | -10.91 | 15.0% | 4 | 2.73 | **0.30** |
| *sum:* | - | *-72.97* | *100.0%* | - | - | - |

**NH** - number of non hydrogen (heavy) atoms.
**GE[1]** - group efficiency calculated as a ratio of scoring function value and number of non hydrogen atoms.
**GE[2]** - group efficiency (see *Eq. 4*) calculated as a ratio of the product of fragment share and experimental binding energy (see *Eq. 2*) and number of non hydrogen atoms.
*** E, kcal/mol** = 0.6 × log p

Table S8. Values of scoring function AutoDock 4 obtained by scoring of compound **2** fragments with experimental geometry in the binding sites of target models (PDB:3IVX)

| **Fragment** | **E, kcal/mol** | **Share, %** | **NH** | **GE[1]** | **GE[2]** |
|---|---|---|---|---|---|
| **1** | -3.25 | 29.7% | 9 | 0.36 | **0.26** |
| **2** | -0.88 | 8.0% | 6 | 0.15 | **0.10** |
| **3** | -4.76 | 43.5% | 11 | 0.43 | **0.31** |
| **4** | -2.06 | 18.8% | 4 | 0.52 | **0.37** |
| *sum:* | *-10.95* | *100.0%* | - | - | - |

**NH** - number of non hydrogen (heavy) atoms.
**GE**$^1$ - group efficiency calculated as a ratio of scoring function value and number of non hydrogen atoms.
**GE**$^2$ - group efficiency (see *Eq. 4*) calculated as a ratio of the product of fragment share and experimental binding energy (see *Eq. 2*) and number of non hydrogen atoms.

Table S9. Values of scoring function AutoDock 4 (Vina 1.2) obtained by scoring of compound **2** fragments with experimental geometry in the binding sites of target models (PDB:3IVX)

| Fragment | E, kcal/mol | Share, % | NH | GE$^1$ | GE$^2$ |
|---|---|---|---|---|---|
| **1** | -3.25 | 29.7% | 9 | 0.36 | **0.26** |
| **2** | -0.93 | 8.5% | 6 | 0.16 | **0.11** |
| **3** | -4.70 | 42.9% | 11 | 0.43 | **0.31** |
| **4** | -2.06 | 18.8% | 4 | 0.52 | **0.37** |
| *sum:* | *-10.95* | *100.0%* | - | - | - |

**NH** - number of non hydrogen (heavy) atoms.
**GE**$^1$ - group efficiency calculated as a ratio of scoring function value and number of non hydrogen atoms.
**GE**$^2$ - group efficiency (see *Eq. 4*) calculated as a ratio of the product of fragment share and experimental binding energy (see *Eq. 2*) and number of non hydrogen atoms.

Table S10. Values of scoring function AutoDock Vina 1.1.2 obtained by scoring of compound **2** fragments with experimental geometry in the binding sites of target models (PDB:3IVX)

| Fragment | E, kcal/mol | Share, % | NH | GE$^1$ | GE$^2$ |
|---|---|---|---|---|---|
| **1** | -2.81 | 24.3% | 9 | 0.31 | **0.21** |
| **2** | -1.10 | 9.5% | 6 | 0.18 | **0.12** |
| **3** | -5.41 | 46.7% | 11 | 0.49 | **0.33** |
| **4** | -2.25 | 19.5% | 4 | 0.56 | **0.38** |
| *sum:* | *-11.58* | *100.0%* | - | - | - |

**NH** - number of non hydrogen (heavy) atoms.
**GE**$^1$ - group efficiency calculated as a ratio of scoring function value and number of non hydrogen atoms.
**GE**$^2$ - group efficiency (see *Eq. 4*) calculated as a ratio of the product of fragment share and experimental binding energy (see *Eq. 2*) and number of non hydrogen atoms.

Table S11. Values of scoring function AutoDock Vina 1.2.3 obtained by scoring of compound **2** fragments with experimental geometry in the binding sites of target models (PDB:3IVX)

| Fragment | E, kcal/mol | Share, % | NH | GE$^1$ | GE$^2$ |
|---|---|---|---|---|---|
| **1** | -2.81 | 24.3% | 9 | 0.31 | **0.21** |
| **2** | -1.11 | 9.5% | 6 | 0.18 | **0.12** |

| | | | | | |
|---|---|---|---|---|---|
| **3** | -5.41 | 46.7% | 11 | 0.49 | **0.33** |
| **4** | -2.25 | 19.4% | 4 | 0.56 | **0.38** |
| *sum:* | *-11.58* | *100.0%* | - | - | - |

**NH** - number of non hydrogen (heavy) atoms.
**GE[1]** - group efficiency calculated as a ratio of scoring function value and number of non hydrogen atoms.
**GE[2]** - group efficiency (see *Eq. 4*) calculated as a ratio of the product of fragment share and experimental binding energy (see *Eq. 2*) and number of non hydrogen atoms.

Table S12. Values of scoring function Vinardo (Vina 1.2.3) obtained by scoring of compound **2** fragments with experimental geometry in the binding sites of target models (PDB:3IVX)

| Fragment | E, kcal/mol | Share, % | NH | GE[1] | GE[2] |
|---|---|---|---|---|---|
| **1** | -1.00 | 14.3% | 9 | 0.11 | **0.12** |
| **2** | -0.80 | 11.4% | 6 | 0.13 | **0.15** |
| **3** | -3.23 | 46.4% | 11 | 0.29 | **0.33** |
| **4** | -1.93 | 27.8% | 4 | 0.48 | **0.54** |
| *sum:* | *-6.96* | *100.0%* | - | - | - |

**NH** - number of non hydrogen (heavy) atoms.
**GE[1]** - group efficiency calculated as a ratio of scoring function value and number of non hydrogen atoms.
**GE[2]** - group efficiency (see *Eq. 4*) calculated as a ratio of the product of fragment share and experimental binding energy (see *Eq. 2*) and number of non hydrogen atoms.

Table S13. Values of scoring function ΔVinaRF20 obtained by scoring of compound **2** fragments with experimental geometry in the binding sites of target models (PDB:3IVX)

| Fragment | $pK_d$ | E, kcal/mol* | Share, % | NH | GE[1] | GE[2] |
|---|---|---|---|---|---|---|
| **1** | 1.90 | -2.60 | 24.0% | 9 | 0.29 | **0.21** |
| **2** | 0.94 | -1.29 | 11.9% | 6 | 0.21 | **0.15** |
| **3** | 4.16 | -5.70 | 52.5% | 11 | 0.52 | **0.37** |
| **4** | 0.93 | -1.27 | 11.7% | 4 | 0.32 | **0.23** |
| *sum:* | - | *-10.86* | *100.0%* | - | - | - |

**NH** - number of non hydrogen (heavy) atoms.
**GE[1]** - group efficiency calculated as a ratio of scoring function value and number of non hydrogen atoms.
**GE[2]** - group efficiency (see *Eq. 4*) calculated as a ratio of the product of fragment share and experimental binding energy (see *Eq. 2*) and number of non hydrogen atoms.
*** E, kcal/mol** = $pK_d$/-0.73

Table S14. Values of scoring function DrugScore X obtained by scoring of compound **2** fragments with experimental geometry in the binding sites of target models (PDB:3IVX)

| Fragment | log p | E, kcal/mol* | Share, % | NH | GE[1] | GE[2] |
|---|---|---|---|---|---|---|
| **1** | -29.51 | -17.70 | 23.0% | 9 | 1.97 | **0.20** |
| **2** | -10.93 | -6.56 | 8.5% | 6 | 1.09 | **0.11** |
| **3** | -74.98 | -44.99 | 58.3% | 11 | 4.09 | **0.42** |
| **4** | -13.09 | -7.86 | 10.2% | 4 | 1.96 | **0.20** |
| *sum:* | - | *-77.11* | *100.0%* | - | - | - |

**NH** - number of non hydrogen (heavy) atoms.
**GE[1]** - group efficiency calculated as a ratio of scoring function value and number of non hydrogen atoms.
**GE[2]** - group efficiency (see *Eq. 4*) calculated as a ratio of the product of fragment share and experimental binding energy (see *Eq. 2*) and number of non hydrogen atoms.
*** E, kcal/mol** = 0.6 × log p

Table S15. Values of scoring function AutoDock 4 obtained by scoring of compound **1** fragments with docked geometry in the binding site of target models (PDB: 3IMC)

| Fragment | E, kcal/mol | Share, % | NH | GE[1] | GE[2] |
|---|---|---|---|---|---|
| **1** | -1.64 | 17.6% | 7 | 0.23 | **0.20** |
| **2** | -0.90 | 9.7% | 6 | 0.15 | **0.13** |
| **3** | -4.32 | 46.4% | 11 | 0.39 | **0.33** |
| **4** | -2.45 | 26.3% | 4 | 0.61 | **0.52** |
| *sum:* | *-9.31* | *100.0%* | - | - | - |

**NH** - number of non hydrogen (heavy) atoms.
**GE[1]** - group efficiency calculated as a ratio of scoring function value and number of non hydrogen atoms.
**GE[2]** - group efficiency (see *Eq. 4*) calculated as a ratio of the product of fragment share and experimental binding energy (see *Eq. 2*) and number of non hydrogen atoms.

Table S16. Values of scoring function AutoDock 4 (Vina 1.2) obtained by scoring of compound **1** fragments with docked geometry in the binding sites of target models (PDB:3IMC)

| Fragment | E, kcal/mol | Share, % | NH | GE[1] | GE[2] |
|---|---|---|---|---|---|
| **1** | -1.64 | 17.6% | 7 | 0.23 | **0.20** |
| **2** | -0.95 | 10.3% | 6 | 0.16 | **0.14** |
| **3** | -4.25 | 45.8% | 11 | 0.39 | **0.33** |
| **4** | -2.45 | 26.3% | 4 | 0.61 | **0.52** |
| *sum:* | *-9.29* | *100.0%* | - | - | - |

**NH** - number of non hydrogen (heavy) atoms.

**GE¹** - group efficiency calculated as a ratio of scoring function value and number of non hydrogen atoms.
**GE²** - group efficiency (see *Eq. 4*) calculated as a ratio of the product of fragment share and experimental binding energy (see *Eq. 2*) and number of non hydrogen atoms.

Table S17. Values of scoring function AutoDock Vina 1.1.2 obtained by scoring of compound **1** fragments with docked geometry in the binding sites of target models (PDB:3IMC)

| Fragment | E, kcal/mol | Share, % | NH | GE¹ | GE² |
|---|---|---|---|---|---|
| **1** | -1.66 | 14.1% | 7 | 0.24 | **0.16** |
| **2** | -2.35 | 20.0% | 6 | 0.39 | **0.26** |
| **3** | -5.53 | 47.0% | 11 | 0.50 | **0.34** |
| **4** | -2.21 | 18.8% | 4 | 0.55 | **0.37** |
| *sum:* | *-11.76* | *100.0%* | - | - | - |

**NH** - number of non hydrogen (heavy) atoms.
**GE¹** - group efficiency calculated as a ratio of scoring function value and number of non hydrogen atoms.
**GE²** - group efficiency (see *Eq. 4*) calculated as a ratio of the product of fragment share and experimental binding energy (see *Eq. 2*) and number of non hydrogen atoms.

Table S18. Values of scoring function AutoDock Vina 1.2.3 obtained by scoring of compound **1** fragments with docked geometry in the binding sites of target models (PDB:3IMC)

| Fragment | E, kcal/mol | Share, % | NH | GE¹ | GE² |
|---|---|---|---|---|---|
| **1** | -1.66 | 14.1% | 7 | 0.24 | **0.16** |
| **2** | -2.36 | 20.0% | 6 | 0.39 | **0.27** |
| **3** | -5.53 | 47.0% | 11 | 0.50 | **0.34** |
| **4** | -2.21 | 18.8% | 4 | 0.55 | **0.37** |
| *sum:* | *-11.75* | *100.0%* | - | - | - |

**NH** - number of non hydrogen (heavy) atoms.
**GE¹** - group efficiency calculated as a ratio of scoring function value and number of non hydrogen atoms.
**GE²** - group efficiency (see *Eq. 4*) calculated as a ratio of the product of fragment share and experimental binding energy (see *Eq. 2*) and number of non hydrogen atoms.

Table S19. Values of scoring function Vinardo (Vina 1.2.3) obtained by scoring of compound **1** fragments with docked geometry in the binding sites of target models (PDB:3IMC)

| Fragment | E, kcal/mol | Share, % | NH | GE¹ | GE² |
|---|---|---|---|---|---|
| **1** | -0.98 | 14.4% | 7 | 0.14 | **0.16** |
| **2** | -0.95 | 14.0% | 6 | 0.16 | **0.19** |
| **3** | -3.22 | 47.3% | 11 | 0.29 | **0.34** |

| | | | | | |
|---|---|---|---|---|---|
| **4** | -1.66 | 24.3% | 4 | 0.41 | **0.48** |
| *sum:* | *-6.81* | *100.0%* | - | - | - |

**NH** - number of non hydrogen (heavy) atoms.
**GE**$^1$ - group efficiency calculated as a ratio of scoring function value and number of non hydrogen atoms.
**GE**$^2$ - group efficiency (see *Eq. 4*) calculated as a ratio of the product of fragment share and experimental binding energy (see *Eq. 2*) and number of non hydrogen atoms.

Table S20. Values of scoring function ΔVinaRF20 obtained by scoring of compound **1** fragments with docked geometry in the binding sites of target models (PDB:3IMC)

| Fragment | $pK_d$ | E, kcal/mol* | Share, % | NH | GE$^1$ | GE$^2$ |
|---|---|---|---|---|---|---|
| **1** | 1.69 | -2.32 | 19.3% | 7 | 0.33 | **0.22** |
| **2** | 1.85 | -2.53 | 21.1% | 6 | 0.42 | **0.28** |
| **3** | 4.24 | -5.81 | 48.5% | 11 | 0.53 | **0.35** |
| **4** | 0.97 | -1.33 | 11.1% | 4 | 0.33 | **0.22** |
| *sum:* | - | *-11.99* | *100.0%* | - | - | - |

**NH** - number of non hydrogen (heavy) atoms.
**GE**$^1$ - group efficiency calculated as a ratio of scoring function value and number of non hydrogen atoms.
**GE**$^2$ - group efficiency (see *Eq. 4*) calculated as a ratio of the product of fragment share and experimental binding energy (see *Eq. 2*) and number of non hydrogen atoms.
*** E, kcal/mol** = $pK_d/-0.73$

Table S21. Values of scoring function DrugScore X obtained by scoring of compound **1** fragments with docked geometry in the binding sites of target models (PDB:3IMC)

| Fragment | log p | E, kcal/mol* | Share, % | NH | GE$^1$ | GE$^2$ |
|---|---|---|---|---|---|---|
| **1** | -15.58 | -9.35 | 15.1% | 7 | 1.34 | **0.17** |
| **2** | -9.68 | -5.81 | 9.4% | 6 | 0.97 | **0.12** |
| **3** | -69.86 | -41.92 | 67.6% | 11 | 3.81 | **0.49** |
| **4** | -8.20 | -4.92 | 7.9% | 4 | 1.23 | **0.16** |
| *sum:* | - | *-61.99* | *100.0%* | - | - | - |

**NH** - number of non hydrogen (heavy) atoms.
**GE**$^1$ - group efficiency calculated as a ratio of scoring function value and number of non hydrogen atoms.
**GE**$^2$ - group efficiency (see *Eq. 4*) calculated as a ratio of the product of fragment share and experimental binding energy (see *Eq. 2*) and number of non hydrogen atoms.
*** E, kcal/mol** = 0.6 × log p

Table S22. Values of scoring function AutoDock 4 obtained by scoring of compound **2** fragments with docked geometry in the binding sites of target models (PDB:3IMC)

| Fragment | E, kcal/mol | Share, % | NH | GE[1] | GE[2] |
|---|---|---|---|---|---|
| **1** | -3.80 | 32.9% | 9 | 0.42 | **0.29** |
| **2** | -1.12 | 9.7% | 6 | 0.19 | **0.13** |
| **3** | -3.43 | 29.7% | 11 | 0.31 | **0.21** |
| **4** | -3.21 | 27.8% | 4 | 0.80 | **0.54** |
| *sum:* | *-11.56* | *100.0%* | - | - | - |

**NH** - number of non hydrogen (heavy) atoms.
**GE[1]** - group efficiency calculated as a ratio of scoring function value and number of non hydrogen atoms.
**GE[2]** - group efficiency (see *Eq. 4*) calculated as a ratio of the product of fragment share and experimental binding energy (see *Eq. 2*) and number of non hydrogen atoms.

Table S23. Values of scoring function AutoDock 4 (Vina 1.2) obtained by scoring of compound **2** fragments with docked geometry in the binding sites of target models (PDB:3IMC)

| Fragment | E, kcal/mol | Share, % | NH | GE[1] | GE[2] |
|---|---|---|---|---|---|
| **1** | -3.80 | 33.0% | 9 | 0.42 | **0.29** |
| **2** | -1.14 | 9.9% | 6 | 0.19 | **0.13** |
| **3** | -3.37 | 29.3% | 11 | 0.31 | **0.21** |
| **4** | -3.21 | 27.9% | 4 | 0.80 | **0.55** |
| *sum:* | *-11.52* | *100.0%* | - | - | - |

**NH** - number of non hydrogen (heavy) atoms.
**GE[1]** - group efficiency calculated as a ratio of scoring function value and number of non hydrogen atoms.
**GE[2]** - group efficiency (see *Eq. 4*) calculated as a ratio of the product of fragment share and experimental binding energy (see *Eq. 2*) and number of non hydrogen atoms.

Table S24. Values of scoring function AutoDock Vina 1.1.2 obtained by scoring of compound **2** fragments with docked geometry in the binding sites of target models (PDB:3IMC)

| Fragment | E, kcal/mol | Share, % | NH | GE[1] | GE[2] |
|---|---|---|---|---|---|
| **1** | -4.40 | 32.5% | 9 | 0.49 | **0.28** |
| **2** | -2.23 | 16.5% | 6 | 0.37 | **0.22** |
| **3** | -4.96 | 36.7% | 11 | 0.45 | **0.26** |
| **4** | -1.94 | 14.3% | 4 | 0.48 | **0.28** |
| *sum:* | *-13.53* | *100.0%* | - | - | - |

**NH** - number of non hydrogen (heavy) atoms.
**GE[1]** - group efficiency calculated as a ratio of scoring function value and number of non hydrogen atoms.

**GE²** - group efficiency (see *Eq. 4*) calculated as a ratio of the product of fragment share and experimental binding energy (see *Eq. 2*) and number of non hydrogen atoms.

Table S25. Values of scoring function AutoDock Vina 1.2.3 obtained by scoring of compound **2** fragments with docked geometry in the binding sites of target models (PDB:3IMC)

| Fragment | E, kcal/mol | Share, % | NH | GE¹ | GE² |
|---|---|---|---|---|---|
| **1** | -4.40 | 32.5% | 9 | 0.49 | **0.28** |
| **2** | -2.23 | 16.5% | 6 | 0.37 | **0.22** |
| **3** | -4.95 | 36.6% | 11 | 0.45 | **0.26** |
| **4** | -1.94 | 14.3% | 4 | 0.49 | **0.28** |
| *sum:* | *-13.52* | *100.0%* | - | - | - |

**NH** - number of non hydrogen (heavy) atoms.
**GE¹** - group efficiency calculated as a ratio of scoring function value and number of non hydrogen atoms.
**GE²** - group efficiency (see *Eq. 4*) calculated as a ratio of the product of fragment share and experimental binding energy (see *Eq. 2*) and number of non hydrogen atoms.

Table S26. Values of scoring function Vinardo (Vina 1.2.3) obtained by scoring of compound **2** fragments with docked geometry in the binding sites of target models (PDB:3IMC)

| Fragment | E, kcal/mol | Share, % | NH | GE¹ | GE² |
|---|---|---|---|---|---|
| **1** | -3.36 | 40.1% | 9 | 0.37 | **0.35** |
| **2** | -1.05 | 12.6% | 6 | 0.18 | **0.16** |
| **3** | -2.35 | 28.0% | 11 | 0.21 | **0.20** |
| **4** | -1.63 | 19.4% | 4 | 0.41 | **0.38** |
| *sum:* | *-8.39* | *100.0%* | - | - | - |

**NH** - number of non hydrogen (heavy) atoms.
**GE¹** - group efficiency calculated as a ratio of scoring function value and number of non hydrogen atoms.
**GE²** - group efficiency (see *Eq. 4*) calculated as a ratio of the product of fragment share and experimental binding energy (see *Eq. 2*) and number of non hydrogen atoms.

Table S27. Values of scoring function ΔVinaRF20 obtained by scoring of compound **2** fragments with docked geometry in the binding sites of target models (PDB:3IMC)

| Fragment | $pK_d$ | E, kcal/mol* | Share, % | NH | GE¹ | GE² |
|---|---|---|---|---|---|---|
| **1** | 3.18 | -4.36 | 32.4% | 9 | 0.48 | **0.28** |
| **2** | 1.76 | -2.41 | 17.9% | 6 | 0.40 | **0.23** |
| **3** | 3.81 | -5.22 | 38.8% | 11 | 0.47 | **0.28** |
| **4** | 1.06 | -1.45 | 10.8% | 4 | 0.36 | **0.21** |

| | | | | | | |
|---|---|---|---|---|---|---|
| *sum:* | - | *-13.44* | *100.0%* | - | - | - |

**NH** - number of non hydrogen (heavy) atoms.
**GE¹** - group efficiency calculated as a ratio of scoring function value and number of non hydrogen atoms.
**GE²** - group efficiency (see *Eq. 4*) calculated as a ratio of the product of fragment share and experimental binding energy (see *Eq. 2*) and number of non hydrogen atoms.
*** E, kcal/mol** = $pK_d$/-0.73

Table S28. Values of scoring function DrugScore X obtained by scoring of compound **1** fragments with docked geometry in the binding sites of target models (PDB:3IMC)

| Fragment | log p | E, kcal/mol* | Share, % | NH | GE¹ | GE² |
|---|---|---|---|---|---|---|
| **1** | -47.94 | -28.76 | 40.4% | 9 | 3.20 | **0.35** |
| **2** | -1.41 | -0.85 | 1.2% | 6 | 0.14 | **0.02** |
| **3** | -58.89 | -35.33 | 49.6% | 11 | 3.21 | **0.35** |
| **4** | -10.48 | -6.29 | 8.8% | 4 | 1.57 | **0.17** |
| *sum:* | - | *-71.23* | *100.0%* | - | - | - |

**NH** - number of non hydrogen (heavy) atoms.
**GE¹** - group efficiency calculated as a ratio of scoring function value and number of non hydrogen atoms.
**GE²** - group efficiency (see *Eq. 4*) calculated as a ratio of the product of fragment share and experimental binding energy (see *Eq. 2*) and number of non hydrogen atoms.
*** E, kcal/mol** = 0.6 × log p

Table S29. The pivot table of group efficiency ($GE^2$) calculated from values of scoring functions obtained by scoring of compound **1** fragments with experimental geometry in the binding sites of target models (PDB:3IUE)

| Fragment | AD4 | ADV | Vinardo | ΔVinaRF20 | DSX | Mean GE | Std. Deviation |
|---|---|---|---|---|---|---|---|
| **1** | 0.18 | 0.20 | 0.16 | 0.20 | 0.15 | 0.18 | 0.02 |
| **2** | 0.20 | 0.25 | 0.19 | 0.27 | 0.13 | 0.21 | 0.05 |
| **3** | 0.32 | 0.33 | 0.31 | 0.34 | 0.45 | 0.35 | 0.06 |
| **4** | 0.50 | 0.37 | 0.57 | 0.29 | 0.30 | 0.40 | 0.12 |

**GE²** - group efficiency (see *Eq. 4*) calculated as a ratio of the product of fragment share and experimental binding energy (see *Eq. 2*) and number of non hydrogen atoms.
**AD4** - AutoDock 4
**ADV** - AutoDock Vina 1.2.3

Table S30. The pivot table of group efficiency ($GE^2$) calculated from values of scoring functions obtained by scoring of compound **2** fragments with experimental geometry in the binding sites of target models (PDB:3IVX)

| Fragment | AD4 | ADV | Vinardo | ΔVinaRF20 | DSX | Mean GE | Std. Deviation |
|---|---|---|---|---|---|---|---|
| **1** | 0.26 | 0.21 | 0.12 | 0.21 | 0.20 | 0.20 | 0.05 |
| **2** | 0.10 | 0.12 | 0.15 | 0.15 | 0.11 | 0.13 | 0.02 |
| **3** | 0.31 | 0.33 | 0.33 | 0.37 | 0.42 | 0.35 | 0.04 |
| **4** | 0.37 | 0.38 | 0.54 | 0.23 | 0.20 | 0.34 | 0.14 |

**GE$^2$** - group efficiency (see *Eq. 4*) calculated as a ratio of the product of fragment share and experimental binding energy (see *Eq. 2*) and number of non hydrogen atoms.
**AD4** - AutoDock 4
**ADV** - AutoDock Vina 1.2.3

Table S31. The pivot table of group efficiency (GE$^2$) calculated from values of scoring functions obtained by scoring of compound **1** fragments with docked geometry in the binding sites of target models (PDB:3IMC)

| Fragment | AD4 | ADV | Vinardo | ΔVinaRF20 | DSX | Mean GE | Std. Deviation |
|---|---|---|---|---|---|---|---|
| **1** | 0.20 | 0.16 | 0.16 | 0.22 | 0.17 | 0.18 | 0.03 |
| **2** | 0.13 | 0.27 | 0.19 | 0.28 | 0.12 | 0.20 | 0.07 |
| **3** | 0.33 | 0.34 | 0.34 | 0.35 | 0.49 | 0.37 | 0.07 |
| **4** | 0.52 | 0.37 | 0.48 | 0.22 | 0.16 | 0.35 | 0.16 |

**GE$^2$** - group efficiency (see *Eq. 4*) calculated as a ratio of the product of fragment share and experimental binding energy (see *Eq. 2*) and number of non hydrogen atoms.
**AD4** - AutoDock 4
**ADV** - AutoDock Vina 1.2.3

Table S32. The pivot table of group efficiency (GE$^2$) calculated from values of scoring functions obtained by scoring of compound **2** fragments with docked geometry in the binding sites of target models (PDB:3IMC)

| Fragment | AD4 | ADV | Vinardo | ΔVinaRF20 | DSX | Mean GE | Std. Deviation |
|---|---|---|---|---|---|---|---|
| **1** | 0.29 | 0.28 | 0.35 | 0.28 | 0.35 | 0.31 | 0.04 |
| **2** | 0.13 | 0.22 | 0.16 | 0.23 | 0.02 | 0.15 | 0.09 |
| **3** | 0.21 | 0.26 | 0.20 | 0.28 | 0.35 | 0.26 | 0.06 |
| **4** | 0.54 | 0.28 | 0.38 | 0.21 | 0.17 | 0.32 | 0.15 |

**GE$^2$** - group efficiency (see *Eq. 4*) calculated as a ratio of the product of fragment share and experimental binding energy (see *Eq. 2*) and number of non hydrogen atoms.
**AD4** - AutoDock 4
**ADV** - AutoDock Vina 1.2.3
